# Retraction Note to: A Comparative Study of the Toxicity of Polyethylene Glycol–Coated Cobalt Ferrite Nanospheres and Nanoparticles

**DOI:** 10.1186/s11671-020-03441-7

**Published:** 2020-11-05

**Authors:** Shahnaz Akhtar, Qasim Khan, Shahzad Anwar, Ghafar Ali, Muhammad Maqbool, Maaz Khan, Shafqat Karim, Lan Gao

**Affiliations:** 1 School of Life Sciences, Lanzhou University, Lanzhou, 730000 Gansu People’s Republic of China; 2 Shenzhen Key Laboratory of Flexible Memory Materials and Devices, College of Electronic Science and Technology, Shenzhen University, Shenzhen, 518000 People’s Republic of China; 3 Department of Physics, Islamia College Peshawar (Chartered University), Peshawar, 25120 Pakistan; 4 Nanomaterials Research Group, Physics Division PINSTECH, Nilore, Islamabad 45650 Pakistan; 5 Department of Clinical & Diagnostic Sciences, The University of Alabama at Birmingham, Birmingham, AL 35294-1212 USA

## Retraction Note to: Nanoscale Research Letters (2019) 14:386 10.1186/s11671-019-3202-9

The authors have retracted their article [1] because a number of the panels in Figs. 6 and 7 overlap with panels in figures in previously published articles [2, 3], specifically:

- Figure 6 Liver control [1] appears identical to Fig. 10 liver control [2]
- Figure 6 Lung PEG-NPs [1] appears identical to Fig. 10 lung PEG-CF [2]
- Figure 6 Spleen control [1] appears identical to Fig. 10 spleen control [2]
- Figure 6 Spleen treatment [1] appears identical to Fig. 10 spleen treatment [2]
- Figure 7A [1] appears to be identical but in a different order to Fig. 6A all panels [3]
- Figure 7B [1] appears identical to Fig. 6B [3]
- Figure 7D [1] appears identical to Fig. 6D [3] using a different scale
- Figure 7C [1] appears identical to Fig. 6E [3] using a different scale

All authors agree with this retraction.
